# Comprehensive transcriptome analysis of sika deer antler using PacBio and Illumina sequencing

**DOI:** 10.1038/s41598-022-20244-1

**Published:** 2022-09-28

**Authors:** Ranran Zhang, Yimeng Dong, Xiumei Xing

**Affiliations:** 1 Institute of Special Animal and Plant Sciences, Chinese Academy of Agricultural Sciences, Changchun, China; 2 Key Laboratory of Genetics, Breeding and Reproduction of Special Economic Animal, Ministry of Agriculture and Rural Affairs of the People’s Republic of China, Changchun, China

**Keywords:** Transcriptomics, Gene expression

## Abstract

Antler is the fastest growing and ossifying tissue in animals and it is a valuable model for cartilage/bone development. To understand the molecular mechanisms of chondrogenesis and osteogenesis of antlers, the PacBio Sequel II and Illumina sequencing technology were combined and used to investigate the mRNA expression profiles in antler tip, middle, and base at six different developmental stages, i.e., at 15th, 25th, 45th, 65th, 100th and 130th growth days. Consequently, we identified 24,856 genes (FPKM > 0.1), including 8778 novel genes. Besides, principal component analysis (PCA) revealed a significant separation between the growth stage (25th, 45th and 65th days) and ossification stage (100th and 130th days). *COL2A1* gene was significantly abundant in the growth stage, whereas *S100A7*, *S100A12*, *S100A8,* and *WFDC18* genes were abundant at the ossification stage. Subsequently screened to 14,765 significantly differentially expressed genes (DEGs), WGCNA and GO functional enrichment analyses revealed that genes related to cell division and chondrocyte differentiation were up-regulated, whereas those with steroid hormone-mediated signaling pathways were down-regulated at ossification stages. Additionally, 25 tumor suppressor genes and 11 oncogenes were identified and were predicted to interact with *p53*. Co-regulation of tumor suppressor genes and oncogenes is responsible for the special growth pattern of antlers. Together, we constructed the most complete sika deer antler transcriptome database so far. The database provides data support for subsequent studies on the molecular mechanism of sika deer antler chondrogenesis and osteogenesis.

## Introduction

Antlers are a unique feature of males in the Cervidae family which regenerate annually as per their sexual cycles. Except in genera Rangifer, where it is found in both sexes, the antler is typically a secondary feature of males. Antler is a bony outgrowth of the cranial frontal bones and differs from the keratinous horn of the Bovidae in that horns are not discarded but are annually re-grown. Annual antler regeneration originates from pedicle periosteum cells differentiating into adipocytes, chondrocytes, osteocytes, and neuron-like cells in vitro^1^. Antler bone growth occurs through a combination of modified endochondral ossification within each antler distal tip after cartilage formation, and intramembranous ossification within the shaft which increases antler diameter^2^. Notably, modified endochondral ossification involves extensive resorption of mineralized cartilage by osteoclast or chondroblast cells, followed by secretion of bone matrix by osteoblasts, and subsequent mineralization^3^. The rapid antler growth (up to 2 cm/d) is based on the proliferation and differentiation of reserve mesenchymal cells in the antler tip, which eventually evolve into cartilage tissue. Yu et al. reported that the correlation between antlers and osteosarcoma gene expression profile (r = 0.67 ~ 0.78) is higher than that of antlers and normal bone tissue (r = 0.33 ~ 0.47); they speculated the existence of similar patterns of antler growth and tumorigenesis^4^. Several proto-oncogenes (*FOS*, *FAM83A*, and *REL*) and tumor suppressor genes (*PML*, *TP53* pathway-related, and *ADAMTS* family members) are positively selected in cervids and highly expressed in the antler.

Antler regrowth is regulated by local and systemic factors and is involved in the reproductive cycle. It is well established that testosterone is a major reproductive hormone regulating antler regrowth^5,6^. A high androgen level is necessary for the initiation of antler mineralization, whereas low levels are responsible for antler casting and regeneration. It was previously showed that testosterone can be converted into estradiol or dihydrotestosterone, which might be responsible for antler ossification and desiccation as well as the death of the velvet^7^. Meanwhile, Suttie found a strong positive correlation between antler growth rate and circulating IGF-1 concentration, which interacts with testosterone to stimulate intense antler growth^8^. Moreover, previous studies revealed a series of growth factors and signaling pathways involved in the regulation of antler regrowth, including *IGF-1*, parathyroid hormone-related peptide, retinoid acid, melatonin, and *Wnt* signaling pathway^9,10^.

These features render the antler a valuable model for studying bone growth and mineralization as well as epimorphic regeneration^11^. Therefore, several scientists have focused on exploring molecular regulatory mechanisms of regeneration, rapid growth, osteogenesis, and shedding of antlers using modern biological technologies (including transcriptomics, and proteomics)^12–14^. Furthermore, Xiumei Xing recently assembled a high-quality sika deer whole-genome sequence using combined technologies of SMRT, Illumina sequencing, and Hi-C, which significantly facilitated our work^15^. Antler growth is a classic "S" type curve, and its gene expression pattern undergoes major changes at different stages. Herein, we collected sika deer antlers during six different development periods, the initial growth period (15th and 25th days), rapid growth period (45th and 65th days), and ossification period (100th and 130th days). Subsequently, a gene expression database was constructed using transcriptomic technology. Meanwhile, we analyzed the gene expression dynamics and screened gene or signaling pathways related to antler chondrogenesis and osteogenesis. Our findings will improve the understanding of the biological processes for antler development at a molecular level. Antlers can complete the growth of cartilage, bone tissue and mineral accumulation in a short time to achieve rapid ossification. Thus, the exploration of the mechanism of rapid growth and ossification of antler provides a reference for the treatment of cartilage- and bone-related disorders, e.g. osteoporosis, fracture healing.

## Results

### Antler transcriptome sequencing

The RNA obtained from the antlers at growth stages (15, 25, 45, and 65 days) were combined to prepare the SMRTbell library (ANTLER1), and the RNA in ossification stages (100 and 130 days) to prepare another library (ANTLER2). We obtained 31.39 Gb of raw data and 14,495,425 sub-reads. The subread BAM of the offline data was self-corrected to obtain 1,367,298 CCS. After clustering the full-length non-chimeric sequences, 252,913 polished consensuses for ANTLER1 and 300,178 for ANTLER2 were generated (Table 1). Corrected consensus sequences were aligned to the sika deer reference genome using the GMAP software, showing a mapping rate of 86.72% and 88.26% for ANTLER1 and ANTLER2 (Table S2). Finally, 86,321 isoforms were generated, of which 14,402 were aligned to unannotated regions of the reference genome GTF file, corresponding to 6593 non-redundant novel genes (Table S3). The IsoSeq cluster representative sequences were added to the reference genome GTF file construct a new reference genome for Illumina short reads alignment and gene expression analysis.

**Table 1:** Data output using the SMRT sequencing platforms.

Statistics	ANTLER1	ANTLER2
Subreads base (G)	14.49	16.9
Subreads numbers	6,838,310	7,657,115
Circular Consensus Sequence	625,715	74,1583
Full-length non-chimeric reads	441,644	545,108
Consensus reads	252,913	300,178
Mean_length of transcripts (bp)	3240	3247
Min_length of transcripts (bp)	170	189
Max_length of transcripts (bp)	17,475	17,839
N50 of transcripts (bp)	4223	4214
N90 of transcripts (bp)	2070	2020

Additionally, 48 samples were analyzed using Illumina technology (Table S1). In total, 2,172,875,780 clean reads were obtained after filtering raw data; the short reads were successfully mapped back to the new reference genome with an average mapping ratio of 90.96%. Genome-mapping results from all the sequenced short-reads data were gathered, assembled using Cufflinks, and compared to known gene models using the Cuffcompare, yielding a total of 2185 novel genes.

### Gene functional annotation

In total, 30,227 genes were functionally annotated against seven public databases (Table 2), of which 21,449 were derived from the reference genome, 6593 were novel genes from Iso-Seq, and 2185 were novel genes from short reads. 28,698 gene sequences (94.94%) were successfully aligned to more than one public database, out of which the NT database had the highest annotation ratio at 93.55%; followed by the NR database with 84.46%. A total of 20,909 genes sequences were annotated in the SwissProt database, corresponding to 17,168 entries.

**Table 2:** Statistics of BLASTX database searches for gene sequences.

Databases	Number of genes	Percentage (%)
NR	25,530	84.46
NT	28,280	93.55
KEGG	13,383	44.27
Swiss-Prot	20,909	69.17
PFAM	18,329	60.63
GO	18,738	61.99
KOG	8965	29.65
In all Databases	5668	18.75
At least one Database	28,698	94.94

### Gene expression analysis

**Figure 1:**
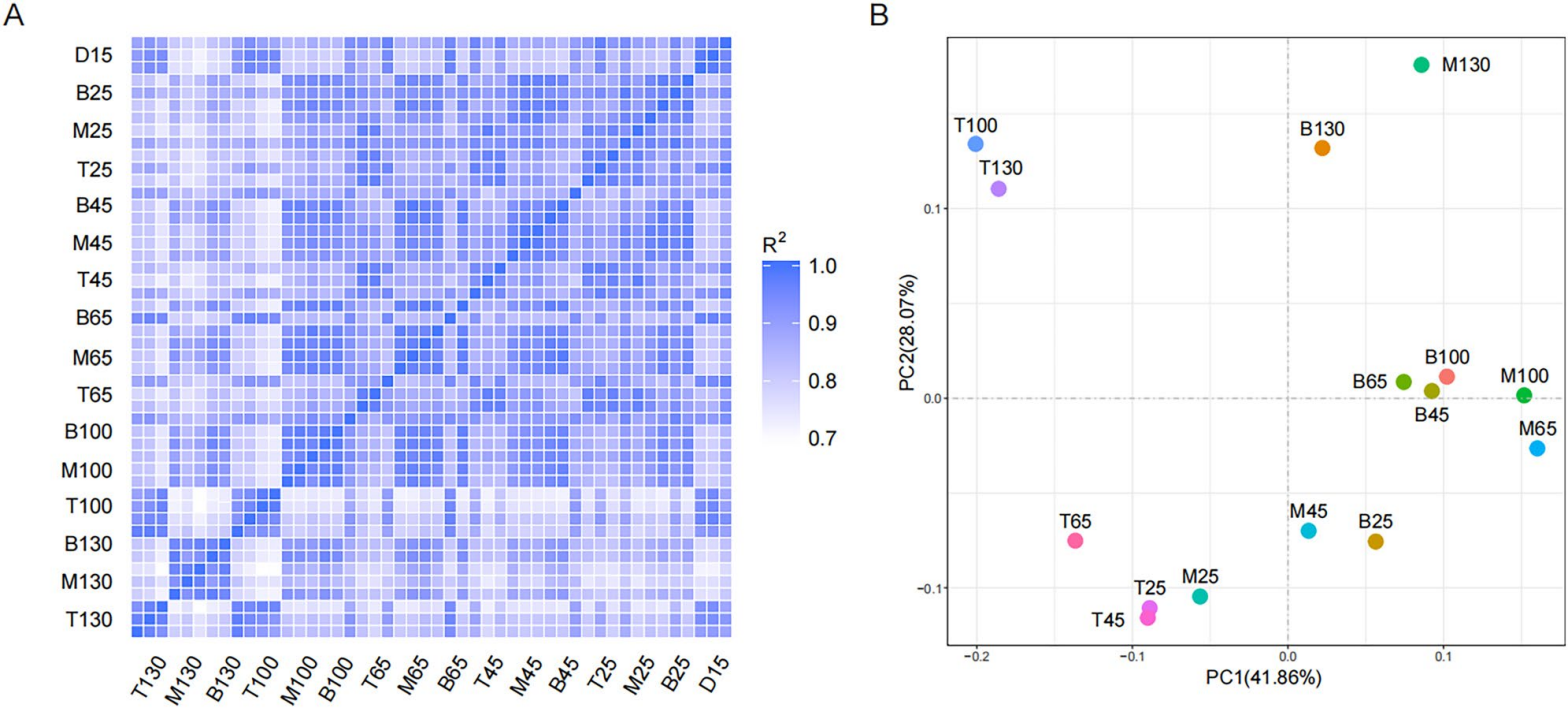
The correlation (**A**) and principal component analysis (**B**) for the antler samples. The figures weres generated using the R software (vesion 4.1.3, https://www.r-project.org/).

First, we performed correlation and principal component analysis (PCA). The squares of Pearson's correlation coefficient (R^2^) were all greater than 0.8, implying that the samples had a good biological repetition (Fig. 1A). PCA analysis revealed that antlers at the growth and ossification periods had a clear separation. Moreover, the antler tip was different from the antler's middle and base (Fig. 1B).

Further, we characterized the 20 most abundant genes per sample (Table S4), covering 51 genes. Among these, Collagen alpha-1(I) chain (*COL1A1*), Collagen alpha-2(I) chain (*COL1A2*), Secreted protein acidic and rich in cysteine (*SPARC*)*,* and Thymosin beta-10 (*TMSB10*) were shared by all samples. Cartilage marker genes (*COL2A1*, *COL3A1*) were specifically prevalent in the top 20 list of T45 and T65, whereas the abundance of Protein S100-A7 (*S100A7*), Protein S100-A12 (*S100A12*), Protein S100-A8 (*S100A8*)*,* and WAP four-disulfide core domain protein 18 (*WFDC18*) drastically increased in T100 and T130. Additionally, several high-abundance genes were related to different parts of the antler. For instance, the expression of osteopontin (*OPN*), Osteocalcin (*OCN*), and pigment epithelium-derived factor (*PEDF*) in the antler middle and base was higher than that in the corresponding antler tip. Although at 45th and 65th days were adjacent periods, the FPKM value of *OCN* of M65 was 20 times that of M45.

### Differentially expressed gene analysis

**Figure 2:**
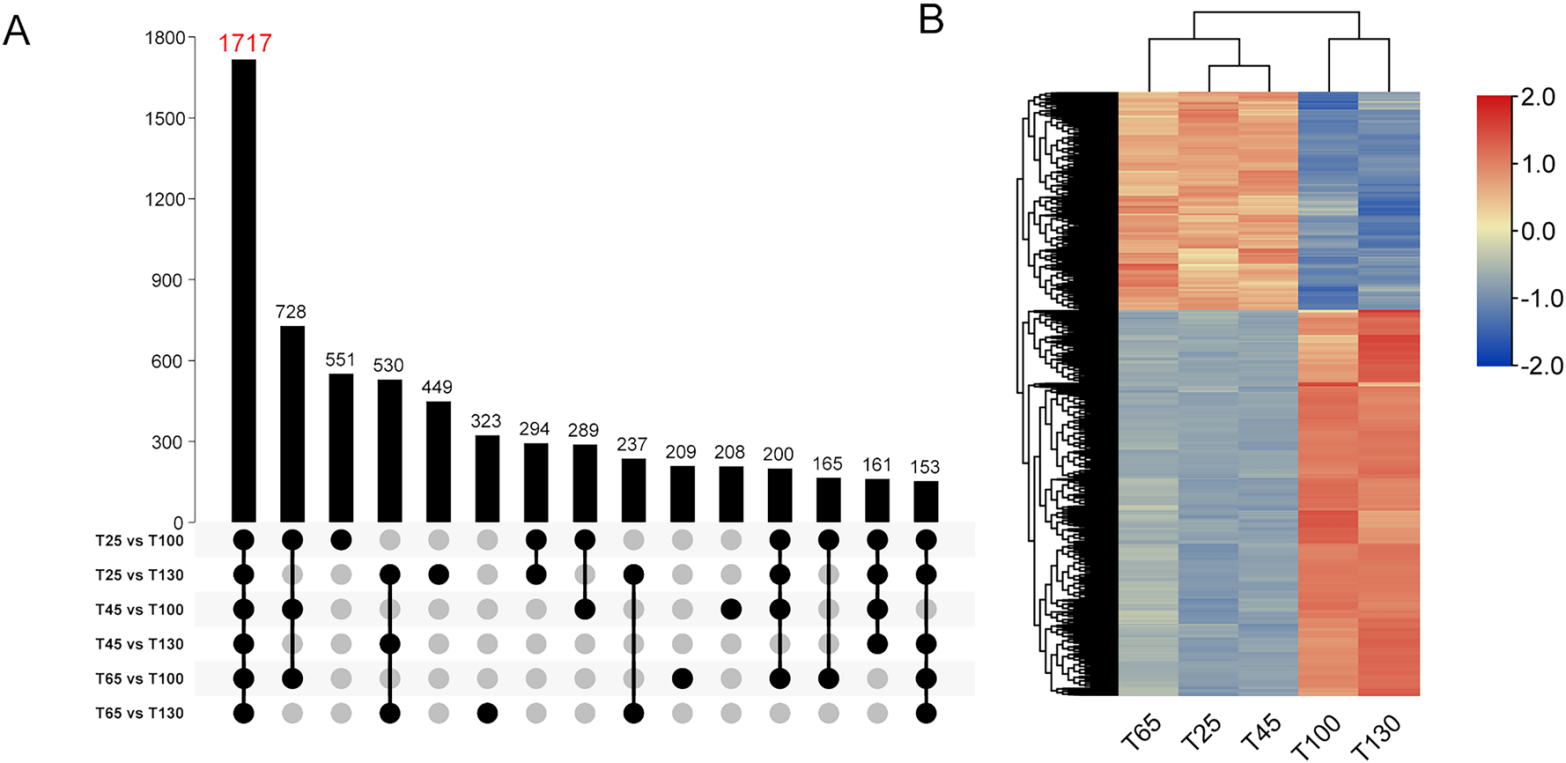
Differentially expressed gene analysis. (**A**) UpSet Plot for DEGs between the growing antler tip and ossification antler tip. 1717 DEGs were shared by the 6 groups. (**B**) Heat map of 1717 differentially expressed genes at growth and ossification stages. The UpSet Plot and gene expression heat map were generated using TBtools software (version 1.082, https://github.com/CJ-Chen/TBtools).

The DESeq2 software was used to screen the significantly differentially expressed genes (DEGs) (*p*-adjusted < 0.05, |log2(foldchange)|> 1) and 14,765 DEGs were obtained. As shown in Table S5, a small number of DEGs were screened in the groups (T25 vs. T45, T25 vs. T65 and T45 vs. T65), which were 8, 7 and 1 respectively. On the contrary, abundant DEGs were discovered between the growing antler tip and ossification antler tip (T25 vs. T100, T25 vs. T130, T45 vs. T100, T45 vs. T130, T65 vs. T100, T65 vs. T130), among which 1717 DEGs were shared by the 6 groups (Fig. 2A). Based on the expression levels of these genes, the antler tip from the five periods could be divided into two clusters, in which T25, T45 and T65 were in one cluster, and T100 and T130 were in the other cluster. Heat map of gene expression showed that 610 genes were up-regualted and 1107 genes were down-regulated in T25, T45 and T65 (Fig. 2B). Gene function enrichment analysis revealed that up-regulated genes were mainly related to collagen fiber assembly, chondrocyte differentiation, osteoblast differentiation, and glycolysis (Table S6). The down-regulated genes were primarily related to the establishment of the skin barrier, arachidonic acid metabolism, innate immune response, and ovarian steroidogenesis. Additionally, the difference between the antler tip and middle gradually increased with the developing antler, whereas the difference between the antler middle and base was smaller.

### WGCNA analysis for DEGs

**Figure 3:**
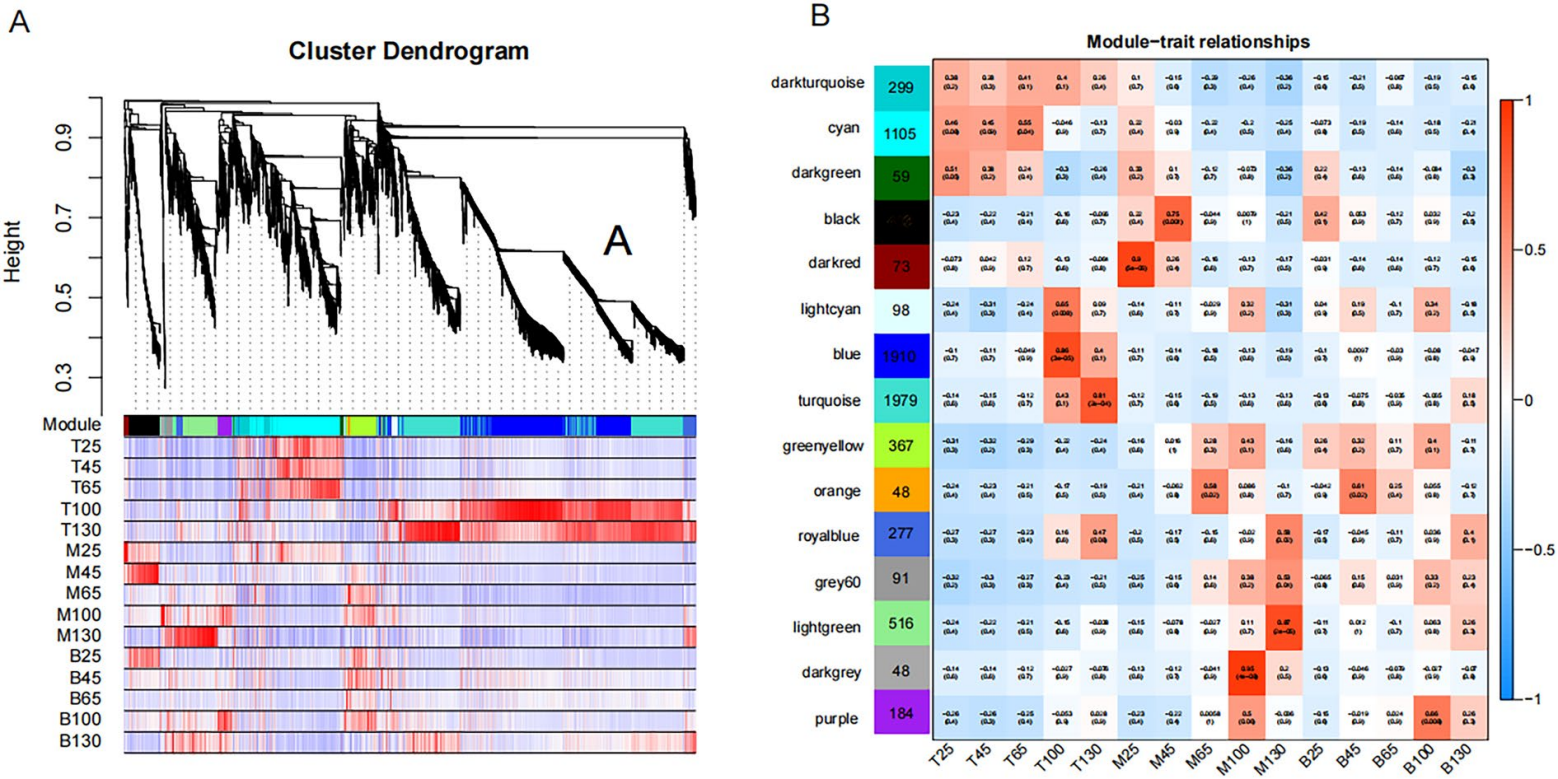
Through WGCNA analysis, 7417 DEGs were divided into 15 distinct modules. (**A**) Gene dendrogram and correlation heat map between gene and module. Red represents positively correlated, blue depicts negatively correlated; (**B**) Correlation heat map between modules and sample. The figures were generated by R WGCNA package (version 1.71, https://cran.r-project.org/package=WGCNA).

**Figure 4:**
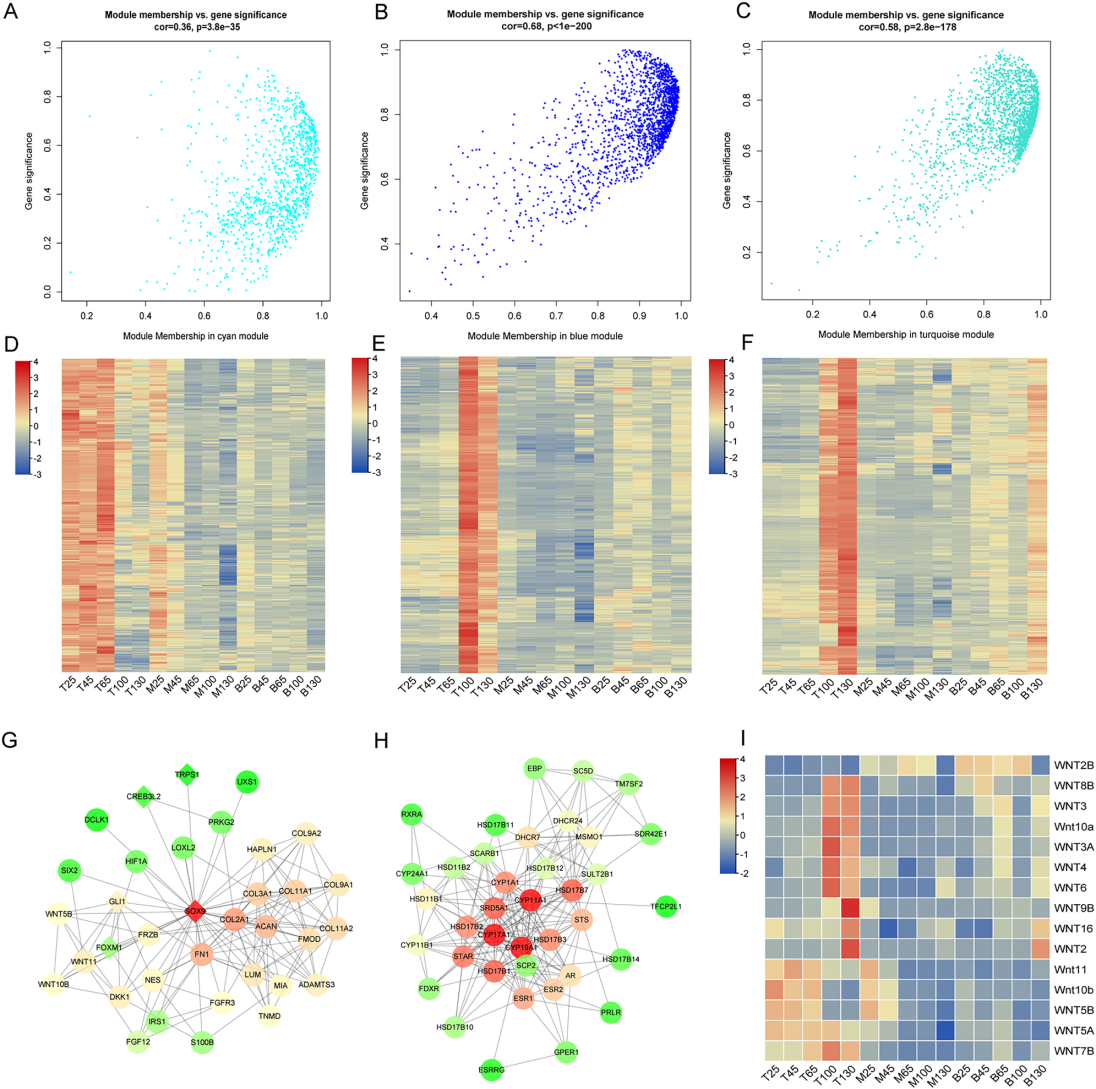
Rapid growth and ossification-related genes and networks. (**A**, **B**, and **C**) Scatter diagram of module membership and gene significance for cyan, blue and turquoise module were obtained by R WGCNA package (https://cran.r-project.org/package=WGCNA); (**D**, **E**, and **F**) Expression heat maps of genes in cyan, blue and turquoise modules were generated using Tbtools software (version 1.082, https://github.com/CJ-Chen/TBtools); (**G** and **E**) Networks of genes associated with rapid growth and ossification were established by the STRING database (version 11.5, https://cn.string-db.org/) and visualized in the Cytoscape software (version 3.4.0, http://www.cytoscape.org/); (**I**) Heat map of 15 WNT gene expression in different antler samples were generated using Tbtools software.

Notably, genes with similar expression patterns participate in similar pathways or have similar functions. Therefore, the weighted gene co-expression network analysis (WGCNA) was used for the modular analysis of DEGs to screen characteristic module genes of different samples (Fig. 3A). Consequently, 7417 DEGs were divided into 15 distinct modules (labeled with different colors in Fig. 3B) and we presented correlation coefficients between the characteristic genes of each module and each different sample. Of note, 3 modules were worthy of special attention, i.e., cyan, blue, and turquoise, which contained 67% of DEGs (Fig. 4A~F). Cyan module comprised 1105 genes, which positively correlated with T25, T45, and T65. Both blue and turquoise modules revealed a positive correlation with the T100 and T130, where the blue module comprised 1910 genes whereas the turquoise module contained 1979 genes.

For further functional enrichment analysis, genes with kME (for modular membership, also known as eigengene-based connectivity) greater than 0.7 were selected as module members. Cyan module contained 874 genes, which were enriched in biological processes including cell division, DNA replication, chondrocyte differentiation, and collagen catabolism (Fig. 4G, Table S7). Blue and Turquoise module genes were enriched in steroid hormone-mediated signaling pathways, retinoic acid receptor signaling pathway, *Wnt* signaling pathway, the establishment of the skin barrier, ceramide biosynthetic process, proteolysis, and retinol metabolic process (Fig. 4H~I, Table S7). Moreover, the Royalblue module revealed a positive correlation with T130, M130, and B130, and the Lightcyan module showed a positive correlation with T100, M100, and B100. These two modules can be used as the characteristic module genes of velvet antler at 130th and 100th growth days, respectively. Besides, special genes at the 130th antler growth day were enriched in translational initiation, immune response, and negative regulation of tumor necrosis factor production. The 100th-day special genes were related to innate immune response and defense response. Other modules comprised fewer genes or were less relevant to specific antler samples.

### Tumor suppressor genes and oncogenes analysis

**Figure 5:**
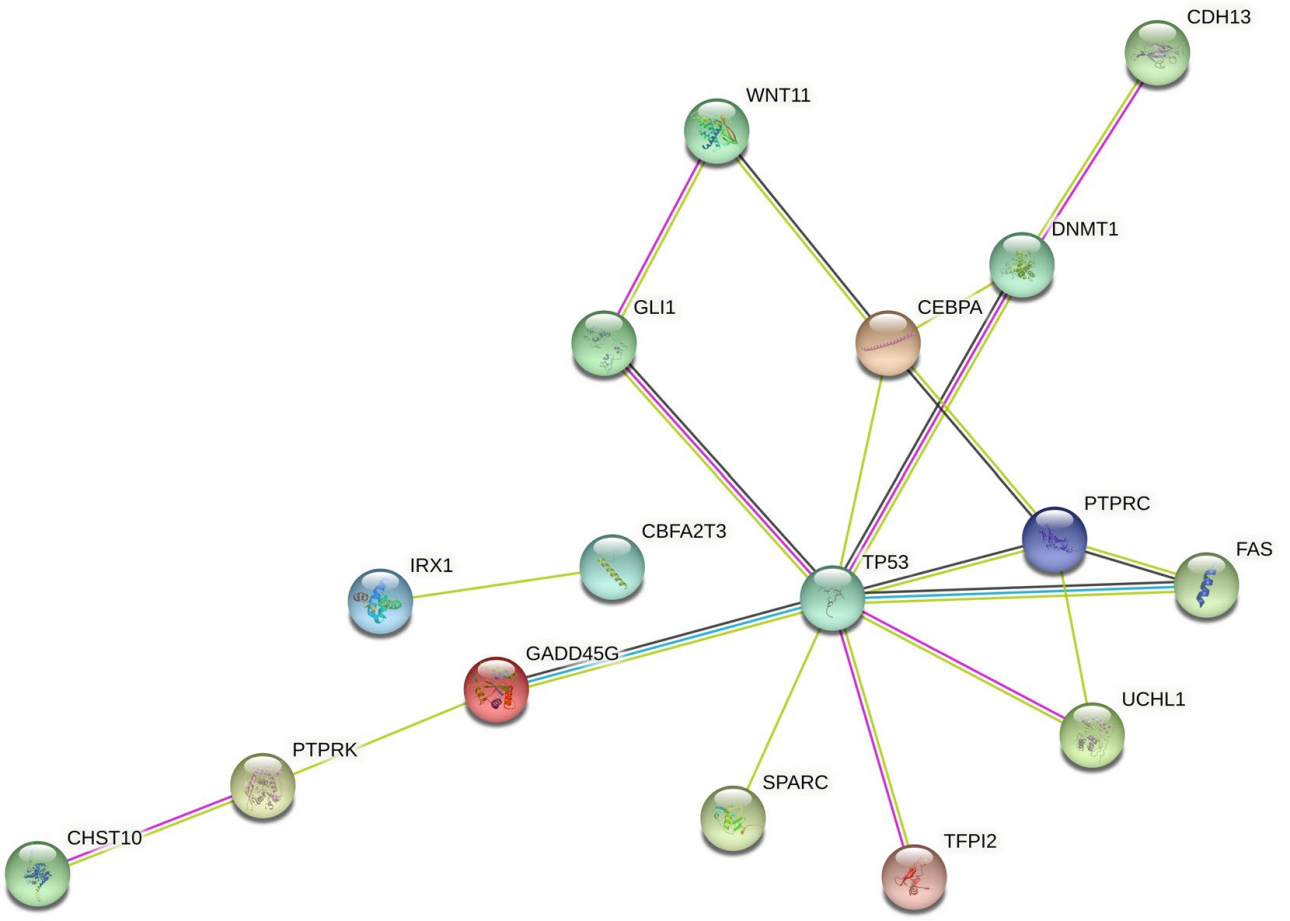
Network of interaction between tumor suppressor and p53 gene. The network was established by the STRING database (version 11.5, https://cn.string-db.org/) and visualized in the Cytoscape software (version 3.4.0, http://www.cytoscape.org/).

Significantly differentially expressed tumor suppressor genes and oncogenes were searched in Tumor Suppressor Gene Database^16^ (https://bioinfo.uth.edu/TSGene/index.html) and Oncogene database (http://ongene.bioinfo-minzhao.org/download.html). A total of 777 differentially expressed tumor suppressor genes and 508 oncogenes were screened, which were involved in cell cycle regulation, apoptosis, cell differentiation, etc. A total of 752 were differentially expressed genes in the antler and osteosarcoma, out of which 490 were also differentially expressed at different growth stages of antler; 25 were included in the list of tumor suppressor genes (Table 3), 11 were included in the list of oncogenes (Table 4). Moreover, STRING online software analysis showed that these genes interact with *TP53*, including *GLI1*, *GADD45G*, *UCHL1*, *FAS*, *DNMT1*, and *PTPRC* (Fig. 5).

**Table 3:** 25 tumor suppressors associated with antler development.

Gene symbol	Gene name	FPKM								
		T25	T45	T65	T100	T130	Osteosarcoma	Osteosarcoma	Roe deer Antler	Sika deer Antler
PIWIL2	Piwi-like protein 2	7.73	7.75	7.90	4.25	3.82	0.34	1.12	7.56	6.59
PTPRK	Receptor-type tyrosine-protein phosphatase kappa	3.86	3.99	3.83	12.43	11.53	0.18	0.09	8.15	6.76
BTG3	Protein BTG3	56.87	57.58	52.51	41.24	37.12	0	0	14.50	20.80
TGM3	Protein-glutamine gamma-glutamyltransferase E	4.50	4.63	10.38	120.73	88.59	2.03	0	14.39	16.21
FOXO6	Forkhead box protein O6	1.11	1.48	2.53	14.28	8.96	0.37	0.1	5.10	4.19
PTPRC	Receptor-type tyrosine-protein phosphatase C	1.80	2.07	1.73	4.03	6.65	0	0.15	7.92	6.90
EPHA1	Ephrin type-A receptor 1	2.40	2.96	6.36	68.56	58.24	3.2	0.03	15.40	11.38
IRX1	Iroquois-class homeodomain protein IRX-1	2.87	2.26	2.35	26.45	17.65	0.44	0.18	13.41	8.64
PLCD1	1-phosphatidylinositol 4,5-bisphosphate phosphodiesterase delta-1	88.64	132.62	98.37	39.61	50.39	9.22	11.59	64.56	67.16
GADD45G	Growth arrest and DNA damage-inducible protein GADD45 gamma	25.56	32.83	50.03	109.59	95.64	6.95	1.5	23.59	32.18
CEBPA	CCAAT/enhancer-binding protein alpha	21.14	20.48	33.60	280.77	170.46	5.25	2.09	38.33	43.15
GLI1	Zinc finger protein GLI1	10.31	9.10	17.96	6.60	1.51	1.09	0.25	3.12	3.15
CHST10	Carbohydrate sulfotransferase 10	96.70	121.45	76.59	31.56	59.25	3.87	4.71	24.00	32.99
FAS	Tumor necrosis factor receptor superfamily member 6	2.48	3.05	3.04	6.73	11.85	8.16	12.28	1.23	0.76
POU2F3	POU domain, class 2, transcription factor 3	2.14	3.66	3.97	66.32	45.87	0.82	0.01	3.08	3.10
PGRMC2	Membrane-associated progesterone receptor component 2	117.91	116.99	109.99	347.47	304.30	16.53	10.72	103.30	118.08
GUCY2C	Heat-stable enterotoxin receptor	0.31	0.28	0.26	1.89	1.28	0.22	0.09	1.15	0.80
TFPI2	Tissue factor pathway inhibitor 2	43.01	31.07	40.06	102.47	260.43	1.08	0.12	50.91	60.36
DNMT1	DNA (cytosine-5)-methyltransferase 1	19.32	18.12	25.10	15.49	12.21	7.08	8.25	41.24	60.53
SPARC	SPARC	14,375.78	14,530.88	10,628.45	6504.71	4483.71	1286.8	1766.17	11,040.98	10,407.78
CDH13	Cadherin-13	24.90	24.07	16.84	55.67	74.93	3.02	5.53	20.53	25.20
ADAMTS18	A disintegrin and metalloproteinase with thrombospondin motifs 18	31.00	36.64	53.94	6.12	1.02	2.99	0.07	23.90	24.05
CBFA2T3	Protein CBFA2T3	3.30	2.71	2.82	7.61	10.14	1.27	0.26	5.20	4.30
WNT11	Protein Wnt-11	23.58	44.15	27.77	9.98	7.47	0.61	0.39	3.96	4.77
UCHL1	Ubiquitin carboxyl-terminal hydrolase isozyme L1	55.84	78.46	186.74	20.74	15.65	5.82	2.91	24.44	26.96

**Table 4:** 11 oncogenes associated with antler development.

Gene symbol	Gene name	FPKM								
		T25	T45	T65	T100	T130	Osteosarcoma	Osteosarcoma	Roe deer Antler	Sika deer Antler
CNTN2	Contactin-2	0.07	0.16	0.19	2.90	0.29	0.05	0.00	0.28	0.31
GLI1	Zinc finger protein GLI1	10.31	9.10	17.96	6.60	1.51	1.09	0.25	3.12	3.15
HMGN5	High mobility group nucleosome-binding domain-containing protein 5	11.66	9.31	9.87	6.01	7.64	5.55	2.11	16.49	14.74
LMO1	Rhombotin-1	1.77	1.38	2.91	30.18	18.24	0.51	0.14	7.60	6.28
MYCN	N-myc proto-oncogene protein	1.89	1.58	1.84	18.04	6.28	0.51	0.10	3.90	3.82
SBSN	Suprabasin	26.06	31.05	66.50	793.69	982.11	0.03	0.00	103.82	113.76
SPARC	SPARC	14,375.78	14,530.88	10,628.45	6504.71	4483.71	1286.80	1766.17	11,040.98	10,407.78
TGM3	Protein-glutamine gamma-glutamyltransferase E	4.50	4.63	10.38	120.73	88.59	2.03	0.00	14.39	16.21
TMEM140	Transmembrane protein 140	4.67	5.22	4.81	8.91	15.60	0.97	0.74	11.73	7.91
UCHL1	Ubiquitin carboxyl-terminal hydrolase isozyme L1	55.84	78.46	186.74	20.74	15.65	5.82	2.91	24.44	26.96
WNT3	Proto-oncogene Wnt-3	1.11	0.93	1.90	24.10	24.99	0.03	0.00	2.75	1.76

### qRT-PCR validation of DEGs

**Figure 6:**
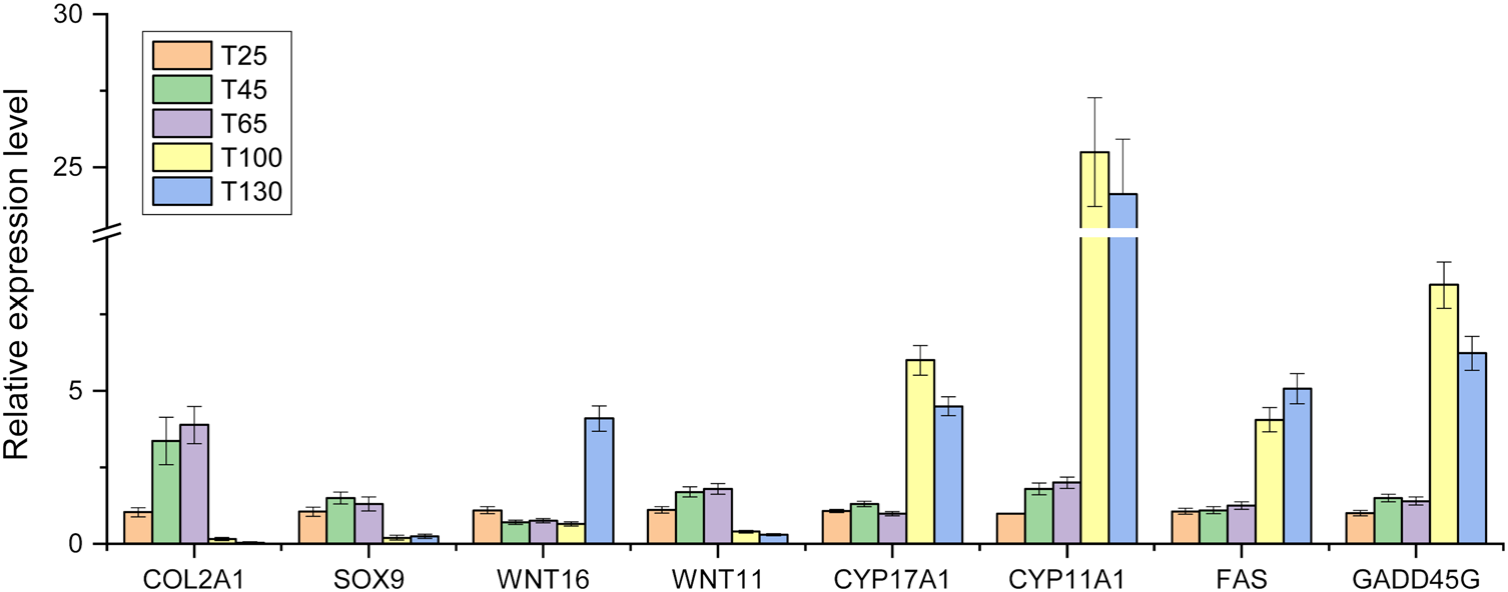
The relative expression levels of 8 genes by qRT-PCR. The figure was generated by OriginPro 2018 software (b9.5.0.193, https://www.originlab.com/).

To further validate the results of RNA-seq, 8 DEGs, including Collagen alpha-1(II) chain (*COL2A1*), Transcription factor SOX-9 (*SOX9*), Protein Wnt-16 (*WNT16*), Protein Wnt-11 (*WNT11*), Steroid 17-alpha-hydroxylase/17,20 lyase (*CYP17A1*), Cholesterol side-chain cleavage enzyme (*CYP11A1*), Tumor necrosis factor receptor superfamily member 6 (*FAS*), Growth arrest and DNA damage-inducible protein GADD45 gamma (*GADD45G*), were selected to perform qRT-PCR. *COL2A1*, *SOX9* and *WNT11* had higher FPKM in the growing stage of antler, while *WNT16*, *CYP17A1*, *CYP11A1*, *FAS* and *GADD45G* had higher FPKM in antler ossifitaion stages. As shown in Fig. 6, the relative expression level of the genes obtained by qRT-PCR were basically consistent with FPKM values by Illumina RNA-seq, which confirmed the reliability of the sequencing data.

## Discussion

The sika deer (*Cervus nippon*) reference genome was adopted in this work, and the RNA-seq mapping ratio was greater than 89%; this was adequate for subsequent analyses. Additionally, we integrated full-length transcriptome analysis, which yielded an additional 6593 non-redundant novel genes; 96.53% of the sequences were annotated in at least one public database. A total of 1343 sequences were blasted to the SwissProt database, corresponding to 398 non-redundant proteins. Higher FPKM values were found in protein *S100-A9*, *WFDC18,* and other genes unidentified in the reference genome. Therefore, the integration of SMRT sequencing further improves the transcriptome expression data of deer antlers.

The dynamic analysis of gene expression showed that the genes related to chondrogenic differentiation had higher FPKM values in the growth stage of antler, this is consistent with the previous proteomics results^14^, including *COL2A1*, *COL11A2*, *ACANA*, and *HAPLN1*. *COL2A1* is a good marker gene for the analysis of molecular events causing initiation and progressive chondrogenesis^17^. *SOX9* is a crucial transcription factor that regulates cartilage development and activates the above-mentioned chondrocyte markers. Baojin Yao discovered that *SOX9* participates in the rapid growth of antlers by controlling multiple cell lineages, including pluripotent mesenchymal cells, proliferating chondrocytes, and hypertrophic chondrocytes^18^. The comparative genomic analysis of ruminants showed that *SOX9* is a highly conserved gene of horned ruminants, involved in the neural crest cell migration pathway and regulates the origin and horn development^4^. Moreover, a headgear-specific gene, *FOXL2*, was upregulated in growing antlers and located upstream of the *SOX9*. This further confirms the important regulatory role of the *SOX9* gene in the rapid growth of the antler. Therefore, the rapid growth of antler may be attributed to the chondrogenesis process mediated by *SOX9*.

In addition to cartilage, bone tissue is also an important component of antler, which is visually demonstrated through the expression of bone matrix proteins (osteopontin and osteocalcin). The former was primarily expressed at the early stage of osteoblast differentiation, and the latter was a late osteoblast differentiation gene promoting bone matrix mineralization^19,20^. Additionally, S100 family genes appear to contribute to osteogenesis, as S100 genes are expressed at higher levels during the ossification phase, including *S100A2*, *S100A4*, *S100A7*, *S100A8*, *S100A9*, *S100A12*, *S100A14*, *S100A15A*, and *S100A16*. Among that, *S100A8* was rich in neutrophils, macrophages, osteoblasts, and osteoclasts. In the presence of Zn^2+^ and Ca^2+^, *S100A8/A9* protein heterodimer participates in cell migration, arachidonic acid metabolism, and bone marrow cell maturation^21^. Also, it increases the calcification of the cartilage matrix and bone trabeculae construction^22^. *TLR4,* a common receptor of *S100A8/A9,* was up-regulated in antlers at the 130th growth day, indicating that *S100A8/A9* may mediate the rapid ossification process of antlers by binding *TLR4*^23,24^.

Then, we performed functional enrichment analysis of the DEGs. The results showed that *Wnt* signaling pathway was an important enrichment pathway, which could mediate distinct effects on the initiation of chondrogenesis and differentiation of chondrocytes^25–28^. For instance, *Wnt5a* and *Wnt5b* promote early chondrogenesis at the same time inhibiting terminal differentiation. In contrast, *Wnt4* suppresses the initiation of chondrogenesis and accelerates terminal chondrocyte differentiation. Besides, *Wnt3a* inhibits chondrogenesis of mesenchymal cells by stabilizing cell–cell adhesion, independent of β-catenin transcriptional activity^29^. Furthermore, *Wnt16* is a vital determinant of cortical bone mass and is majorly derived from osteoblasts. Osteoblast-specific *Wnt16* overexpression increases both cortical and trabecular bone mass and structure in mice^30^. Wergedal discovered that the periosteal bone formation rate and mineral apposition rate were reduced in *Wnt16* knockout vs wild-type mice^31^. Ying Jin revealed that *Wnt16* promotes osteoblast differentiation of periosteal-derived cells in vitro and in vivo with a potential application in improving bone regeneration^32^. Together, it is speculated *Wnt16* signaling pathway can regulate ossification rather than chondrogenesis of antler.

Antler is considered to be a secondary sexual characteristic of male deer, and its periodic regeneration is also associated with sex hormone levels. In this study, it is found that several genes related to steroid hormone mediated signaling pathways were up-regulated during the ossification stage of antler, such as androgen receptor (*AR*), aromatase (*CYP19A1*), steroid 17-alpha-hydroxylase/17,20 lyase (*CYP17A1*), sex hormone-binding globulin (*SHBG*), 3-oxo-5-alpha-steroid 4-dehydrogenase 1 (*SRD5A1*). Androgen regulates bone growth, fracture healing, and bone mass maintenance in male animals. Its receptor (AR) is expressed in mesenchymal cells, osteoblasts, and osteoclasts. Androgen directly binds androgen receptors to regulate bone development in male animals. 5α-reductase, a protein encoded by SRD5A1, could transform testosterone into 5-alpha-dihydrotestosterone (DHT) with more AR binding capacity. 5α-reductase can also be converted into estrogen under the action of P450 aromatase. Therefore, it can be concluded that testosterone play an important role in the ossification of antler. However, the signaling pathway mediated by testosterone still needs to be further explored.

Antler can achieve orderly and rapid growth, and its cell proliferation rate is 30 times faster than cancer cells. Therefore, many scientists have linked the development of antler with cancer, believing that the growth of antler is similar to bone cancer with normal bone growth^33,34^. The significant connection of gene expression between antler and osteosarcoma compared to antler and normal bone helps to explain this viewpoint. Nonetheless, unlike cancerous tissues whose rapid growth is unregulated and pathological, antler tissue regeneration is a highly coordinated physiological process and regulated by multiple tumor suppressors and proto-oncogenes. Based on previous studies, we further screened significantly differentially expressed tumor suppressor genes and oncogenes during antler growth. These genes play important roles in both the initiation and progression of cancer by regulating the cell cycle and apoptosis. For instance, *PLCD1* suppresses the proliferation, invasion, and migration of esophageal squamous cell carcinoma by suppressing the *Wnt/β*-catenin signaling pathway^35^. *GLI1* is a terminal effector of the Hh signaling pathway^36^, which activates numerous downstream proteins, including *SHH*, *PTCH*, and cycle-regulating proteins, including proto-oncogene *N-MYC* and *cycliD*. *DNMT1* is a DNA methyltransferase, whose high expression in tumors is linked to chronic inflammation or persistent viral infection^37^. Additionally, overexpression of these genes including *GLI1*, *GADD45G*, *UCHL1*, *PTPRC*, *DNMT1*, *SPARC*, *TFPI12*, and *FAS* activate transcription of the *p53* gene, which causes apoptosis^38–40^. The *p53* signaling pathway regulates cell division and prevents tumor formation. Yu et al. found that *p53* cofactor genes including *PML*, *NMT2*, and *CD2AP,* and *p53* regulator genes including *ELOVL6*, *S100A8*, *ISG15*, *CNOT3*, and *CCDC69* were expressed in antlers by positive selection of cervid lineage^4^. Summarily, co-regulation of tumor suppressor genes and oncogenes is responsible for special antler growth patterns.

## Conclusion

In conclusion, we combined the PacBio and RNA-seq technologies to analyze the gene expression profiling of antlers during different developmental stages. The results showed that genes associated with chondrogenesis including *COL2A1*, *COL3A1*, *COL11A2*, *ACANA*, *HAPLN1*, and *SOX9* are closely related to the rapid growth process of antler and androgen may promote antler ossification. Moreover, based on previous studies, we further screened the tumor suppressor genes and oncogenes associated with the specific growth pattern of antler. Therefore, our findings provide a reference for subsequent studies on the molecular mechanism of sika deer antler chondrogenesis and osteogenesis.

## Materials and methods

### Samples selection and preparation

Nine 4-year-old male sika deer (*Cervus nippon hortulorum*) was selected from the deer farm of Institute of Special Animal and Plant Sciences, Chinese Academy of Agricultural Sciences. Antlers were harvested on the 15th, 25th, 45th, 65th, 100th, and 130th growth days after casting the previous hard antlers, respectively. Each period had 3 antlers from different deer as repetitions. Therefore, 18 antlers were obtained as samples for this experiment. The deer was first anesthetized with 5% xylazine at 0.5 mg/kg body weight intramuscularly; this was performed according to the ARRIVE guidelines (https://arriveguidelines.org). To remove contaminants from the surface, antlers were washed using PBS buffer. Subsequently, the slices were selected at the tip, middle, and base of antlers, respectively, and ground into powder using the liquid nitrogen grinder. Specifically, the 15-day antler was not segmented and was only used for database construction. All animal experimental protocols were approved and authorized by the Chinese Academy of Agricultural Sciences Animal Care and Use Committee.

### RNA extraction

Total RNA was isolated from antlers using the TRIzol reagent (Invitrogen, CA, USA). RNA degradation and contamination were monitored on 1% agarose gels. Purity, concentration, and integrity were assessed using the NanoPhotometer spectrophotometer (IMPLEN, CA, USA), Qubit 2.0 Fluorimeter (Life Technologies, CA, USA), and Bioanalyzer 2100 system (Agilent Technologies, CA, USA). High-quality RNA was used for transcriptome library construction.

### Transcriptome library construction and sequencing

The Iso-Seq library was prepared as per the Isoform Sequencing protocol (Iso-Seq) using the Clontech SMARTer PCR cDNA Synthesis Kit (Clontech Laboratories, CA, USA) and the BluePippin Size Selection System protocol as described by Pacific Biosciences (PN 100-092-800-03) and sequenced on a PacBio Sequel II platform. The Illumina library was generated using the NEBNext Ultra™ RNA Library Prep Kit for Illumina (NEB, CA, USA) following the manufacturer’s recommendations and sequenced on an Illumina 2500 platform.

### Data processing

Raw Iso-Seq data was processed using the SMRTlink 7.0 software. Circular consensus sequence (CCS) was generated from subread BAM files, parameters: min_length 50, min_passes 1, max_length 15,000. CCS.BAM files were output, which were then classified into full length and non-full length reads using pbclassify.py, ignorepolyA false, minSeqLength 200. Non-full length and full-length fasta files produced were then fed into the cluster step, which does isoform-level clustering (ICE), followed by final Arrow polishing, hq_quiver_min_accuracy 0.99, bin_by_primer false, bin_size_kb 1, qv_trim_5p 100, qv_trim_3p 30. Additional nucleotide errors of SMRT high-quality consensus reads were corrected using the clean Illumina RNA-seq data with the software LoRDEC (vesion 0.7, parameters: k = 23; s = 3).

The GMAP software (version 2017-06-20) was used to align the high-quality polished consensus sequences to the sika deer whole genome sequences (https://bigd.big.ac.cn/gwh; Accession number: GWHANOY00000000) with parameters: no-chimeras; expand-offsets 1; -B 5; -f samse; -n 1. Gene structure analysis was performed using TAPIS (version 1.2.1) pipeline with default parameters. Reads comparable to unannotated regions of the genome were considered novel genes. The short reads were aligned to the reference genome using HISAT software (version 2.0.4) with default parameters.

### Gene functional annotation

All newly predicted genes were annotated using public databases, including the NCBI non-redundant nucleotide database (NT), NCBI non-redundant protein database (NR), Swiss-Prot, Cluster of Orthologous Groups of proteins (COG), and Kyoto Encyclopedia of Genes and Genomes (KEGG)^41^, Protein family (*Pfam*) and Gene Ontology (GO). Further, searches were performed using the BLASTX algorithm with an E-value threshold of 10^−5^.

### Gene expression analysis

Gene expression analysis was performed for each sample using the HTSeq software (vesion 0.6.1; parameters: -m union) with the model union. Statistical analyses of gene expression between different samples were performed according to FPKM. The Pearson correlation coefficients and principal component analysis were calculated and visualized using the R software (vesion 4.1.3). Differential gene expression analysis was performed using the DESeq2 software with screening criteria of foldchange ≥ 2 and *P* value < 0.05. All differential expression genes were analyzed using the WGCNA package of R software (FPKM > 0.1, Module Fold of 0.3, min, ModuleSize of 30, power threshold of 8). GO and KEGG enrichment analyses were conducted using the Tbtools software (version 1.082)^42^. Protein–protein interaction (PPI) network of DEGs was established by the STRING database (version 11.5) and visualized in the Cytoscape software (version 3.4.0). TBtools software (version 1.082) was used to draw UpSet Plot and gene expression heat map of DEGs.

### Quantitative RT-PCR analysis

Quantitative RT-PCR (qRT-PCR) assay was performed using the SYBR® Premix Ex Taq™ kit (TaKaRa, Japan) on a Roche LightCycler480 instrument. The sika deer GAPDH gene was selected as an internal control. Primer sequences used in the experiment are listed in Table S8. Three biological replicates and three technical repeats were used for each gene and sample. The relative mRNA expression level was calculated based on the 2^−ΔΔCT^ method and the figure was drawn with OriginPro 2018.

### Ethical approval

The deer used in this study were approved and authorized by the Chinese Academy of Agricultural Sciences Animal Care and Use Committee. All experimental procedures were carried out in accordance with the approved guidelines and regulations.

## Supplementary Information

Supplementary Information.

## Data Availability

All datasets generated during the current study have been deposited in the NCBI under project number PRJNA831887 (https://www.ncbi.nlm.nih.gov/bioproject/PRJNA831887).

## References

[1] Li C, Yang F, Sheppard A (2009). Adult stem cells and mammalian epimorphic regeneration-insights from studying annual renewal of deer antlers. Curr. Stem Cell Res. Ther..

[2] Price JS, Allen S, Faucheux C, Althnaian T, Mount JG (2005). Deer antlers: A zoological curiosity or the key to understanding organ regeneration in mammals?. J. Anat..

[3] Feleke M (2021). New physiological insights into the phenomena of deer antler: A unique model for skeletal tissue regeneration. J. Orthop. Transl..

[4] Wang Y (2019). Genetic basis of ruminant headgear and rapid antler regeneration. Science.

[5] Gaspar-Lopez E (2010). Biometrics, testosterone, cortisol and antler growth cycle in Iberian red deer stags (Cervus elaphus hispanicus). Reprod. Domest. Anim..

[6] Akhtar RW (2019). Identification of proteins that mediate the role of androgens in antler regeneration using label free proteomics in sika deer (Cervus nippon). Gen. Comp. Endocrinol..

[7] Bubenik GA (2005). Testosterone and estradiol concentrations in serum, velvet skin, and growing antler bone of male white-tailed deer. J. Exp. Zool. A Comp. Exp. Biol..

[8] Suttie JM (1985). Insulin-like growth factor 1 (IGF-1) antler-stimulating hormone?. Endocrinology.

[9] Kierdorf U, Li C, Price JS (2009). Improbable appendages: Deer antler renewal as a unique case of mammalian regeneration. Semin. Cell Dev. Biol..

[10] Sun X (2022). Melatonin promotes antler growth by accelerating MT1-mediated mesenchymal cell differentiation and inhibiting VEGF-induced degeneration of chondrocytes. Int. J. Mol. Sci..

[11] Landete-Castillejos T (2019). Antlers - Evolution, development, structure, composition, and biomechanics of an outstanding type of bone. Bone.

[12] Hu P (2019). Full-length transcriptome and microRNA sequencing reveal the specific gene-regulation network of velvet antler in sika deer with extremely different velvet antler weight. Mol. Genet. Genom..

[13] Sui Z (2020). Quantitative proteomics analysis of deer antlerogenic periosteal cells reveals potential bioactive factors in velvet antlers. J. Chromatogr. A.

[14] Zhang R, Li Y, Xing X (2021). Comparative antler proteome of sika deer from different developmental stages. Sci. Rep..

[15] Xing, X. *et al..* The first high-quality reference genome of sika deer provides insights for high-tannin adaptation (2021). 10.1016/j.gpb.2022.05.008 PMC10372904 35718271

[16] Zhao M, Sun J, Zhao Z (2013). TSGene: A web resource for tumor suppressor genes. Nucleic Acids Res..

[17] Hering TM, Wirthlin L, Ravindran S, McAlinden A (2014). Changes in type II procollagen isoform expression during chondrogenesis by disruption of an alternative 5' splice site within Col2a1 exon 2. Matrix Biol..

[18] Yao B (2018). Sox9 functions as a master regulator of antler growth by controlling multiple cell lineages. DNA Cell Biol..

[19] Paredes R (2004). The Runx2 transcription factor plays a key role in the 1alpha,25-dihydroxy Vitamin D3-dependent upregulation of the rat osteocalcin (OC) gene expression in osteoblastic cells. J. Steroid Biochem. Mol. Biol..

[20] Shen Q, Christakos S (2005). The vitamin D receptor, Runx2, and the Notch signaling pathway cooperate in the transcriptional regulation of osteopontin. J. Biol. Chem..

[21] Goyette J, Geczy CL (2011). Inflammation-associated S100 proteins: New mechanisms that regulate function. Amino Acids.

[22] Zreiqat H, Howlett CR, Gronthos S, Hume D, Geczy CL (2007). S100A8/S100A9 and their association with cartilage and bone. J. Mol. Histol..

[23] Luo G (2020). Bone marrow adipocytes enhance osteolytic bone destruction by activating 1q213(S100A7/8/9-IL6R)-TLR4 pathway in lung cancer. J. Cancer Res. Clin. Oncol..

[24] Ehrchen JM, Sunderkotter C, Foell D, Vogl T, Roth J (2009). The endogenous Toll-like receptor 4 agonist S100A8/S100A9 (calprotectin) as innate amplifier of infection, autoimmunity, and cancer. J. Leukoc Biol..

[25] Ba H, Wang D, Yau TO, Shang Y, Li C (2019). Transcriptomic analysis of different tissue layers in antler growth Center in Sika Deer (Cervus nippon). BMC Genom..

[26] Andrade AC, Nilsson O, Barnes KM, Baron J (2007). Wnt gene expression in the post-natal growth plate: Regulation with chondrocyte differentiation. Bone.

[27] Church V, Nohno T, Linker C, Marcelle C, Francis-West P (2002). Wnt regulation of chondrocyte differentiation. J. Cell Sci..

[28] French DM (2004). WISP-1 is an osteoblastic regulator expressed during skeletal development and fracture repair. Am. J. Pathol..

[29] Hwang SG, Yu SS, Lee SW, Chun JS (2005). Wnt-3a regulates chondrocyte differentiation via c-Jun/AP-1 pathway. FEBS Lett..

[30] Alam I (2016). Osteoblast-specific overexpression of human WNT16 increases both cortical and trabecular bone mass and structure in mice. Endocrinology.

[31] Wergedal JE, Kesavan C, Brommage R, Das S, Mohan S (2015). Role of WNT16 in the regulation of periosteal bone formation in female mice. Endocrinology.

[32] Jin Y, Sun X, Pei F, Zhao Z, Mao J (2020). Wnt16 signaling promotes osteoblast differentiation of periosteal derived cells in vitro and in vivo. PeerJ.

[33] Price J, Allen S (2004). Exploring the mechanisms regulating regeneration of deer antlers. Philos. Trans. R. Soc. Lond. Ser. B Biol. Sci..

[34] Wang DT, Chu WH, Sun HM, Ba HX, Li CY (2017). Expression and functional analysis of tumor-related factor S100A4 in antler stem cells. J. Histochem. Cytochem..

[35] He X (2021). PLCD1 suppressed cellular proliferation, invasion, and migration via inhibition of wnt/beta-catenin signaling pathway in esophageal squamous cell carcinoma. Dig. Dis. Sci..

[36] Lau BW (2019). Hedgehog/GLI1 activation leads to leukemic transformation of myelodysplastic syndrome in vivo and GLI1 inhibition results in antitumor activity. Oncogene.

[37] Kanai Y, Hirohashi S (2007). Alterations of DNA methylation associated with abnormalities of DNA methyltransferases in human cancers during transition from a precancerous to a malignant state. Carcinogenesis.

[38] Yoon JW (2015). p53 modulates the activity of the GLI1 oncogene through interactions with the shared coactivator TAF9. DNA Repair (Amst).

[39] Cai Q (2006). Effects of expression of p53 and Gadd45 on osmotic tolerance of renal inner medullary cells. Am. J. Physiol. Renal. Physiol..

[40] Xiang T (2012). The ubiquitin peptidase UCHL1 induces G0/G1 cell cycle arrest and apoptosis through stabilizing p53 and is frequently silenced in breast cancer. PLoS ONE.

[41] Kanehisa M (2008). KEGG for linking genomes to life and the environment. Nucleic Acids Res..

[42] Chen C (2020). TBtools: An integrative toolkit developed for interactive analyses of big biological data. Mol. Plant.

